# Enhancing collaboration in endometriosis research through the initiative of the World Endometriosis Research Foundation Endometriosis Phenome and Biobanking Harmonisation Project (EPHect)

**DOI:** 10.1093/molehr/gaaf020

**Published:** 2025-07-09

**Authors:** Lone Hummelshoj, Daniëlle Peterse, Kaylon L Bruner-Tran, Stacey A Missmer, Erin Greaves

**Affiliations:** World Endometriosis Research Foundation, London, UK; Vascular Biology Program, Boston Children’s Hospital, Department of Surgery, Harvard Medical School, Boston, MA, USA; World Endometriosis Research Foundation, London, UK; Department of Obstetrics and Gynecology, Vanderbilt University Medical Center, Nashville, TN, USA; World Endometriosis Research Foundation, London, UK; Department of Obstetrics and Gynecology, University of Michigan, Ann Arbor, MI, USA; Department of Epidemiology, Harvard TH Chan School of Public Health, Boston, MA, USA; World Endometriosis Research Foundation, London, UK; Division of Biomedical Sciences, Warwick Medical School, University of Warwick, Coventry, UK

Endometriosis affects an estimated >200 million women (and those assigned female at birth) during their most active and reproductive years, causing a high societal and personal burden (Nnoaham *et al.*, 2011).

It is exactly 100 years since Dr John A. Sampson, a world-renowned gynaecologist of the early 20th century, systematically studied endometriosis and coined its name (Sampson, 1925); however, we still do not know the aetiology, pathophysiology, and natural progression of this disease. Most studies have lacked the detailed phenotypic data necessary to explore heterogeneity among those with endometriosis. Furthermore, discoveries are largely underpowered due to an absence of standardized protocols to robustly compare and accurately meta-analyse results. Consequently, the sparsity of treatments currently available are ‘hit and miss’ in their effectiveness and can have significant, life impacting, side effects (Horne and Missmer, 2022).

We must foster collaboration by working together globally to ensure that research procedures are rigorous, consistent, and informed and influenced by all stakeholders. The World Endometriosis Research Foundation (WERF) Endometriosis Phenome and Biobanking Harmonisation Project (EPHect) was established to facilitate large-scale, international, multi-centre studies that are robust and enable study design and analysis of comprehensive, reproducible, data in a harmonized manner. The ultimate goal is to develop biomarker and treatment targets to advance the diagnosis and management of endometriosis.

In 2014, EPHect published standard tools for the collection of study participant and surgeon-recorded data (Vitonis *et al.*, 2014; Becker *et al.*, 2014) and standard operating procedures (SOPs) for the collection, processing, and storage of tissue and fluid biospecimens (Fassbender *et al.*, 2014; Rahmioglu *et al.*, 2014). A fifth standard tool for physical examination assessment was added in 2024 (Lin *et al.*, 2024).

In this issue of *Molecular Human Reproduction*, we are proud to present an additional four EPHect SOPs for experimental models essential for endometriosis discovery research. Whereas multiple models of endometriosis exist, a lack of harmonization with regard to experimental design, tissue selection, and documentation has impeded reproducibility and direct comparison of results among and between investigators. Herein, we provide comprehensive SOPs to guide and facilitate the choice and implementation of endometriosis models to enhance results interpretation and speed integration and next-stage catalysation. Two sets of SOPs delineate suggestions for unification of *in vivo* mouse models of experimental endometriosis. Specifically, Burns *et al.* (2025) describe the best practices for the establishment of experimental disease using syngeneic mouse endometrium (homologous models) while Hull *et al* (2025) focus on mouse models of disease established using human tissues (heterologous models). A third set of SOPs detail current methods of measuring endometriosis-associated pain in experimental rodent models with recommendations for unification between studies (pain models; Dodds *et al.*, 2025). Finally, we present an SOP for those researchers who prefer an entirely human-based experimental model using matrix-based *in vitro* approaches (organoid models; Marr *et al.*, 2025).

The selection of an appropriate *in vivo* or *in vitro* model to address a particular research question plays a critical role in determining the overall success and reliability of any study. However, this choice is especially significant when investigating a complex and multifactorial condition such as endometriosis. Ideally, a model should mimic the disease manifestation as closely as possible. Currently, however, there is no model available that perfectly replicates the clinical conditions of endometriosis, including the underlying mechanisms and symptoms.

The EPHect Experimental Models Working Group, comprising the efforts of 44 international collaborators from 11 countries representing 5 continents, provides a comprehensive overview of the strengths and limitations of various *in vivo* and *in vitro* models that have been developed to study endometriosis. There are four key determinants that will influence the choice for a particular *in vivo* or *in vitro* model: (i) the specific research question; (ii) the available infrastructure and access to samples within the research environment; (iii) the anticipated timeline to generate the data; and (iv) the available budget to carry out the experiments.

Studying interactions between cells and the *in vivo* environment requires an animal model. Heterologous models are particularly valuable for exploring the disease-associated influence of the original tissue (human) in relation to the surrounding environment (mouse; Hull *et al.*, 2025). Homologous mouse models, on the other hand, are better suited for examining the complexities of the immune system and the influence of specific genes on endometriosis development (Burns *et al.*, 2025). For studies focusing on the effects of a potential novel therapy on endometriosis-associated pain symptoms, a rodent model that allows for behavioural assessments would be the preferred choice (Dodds *et al.*, 2025). Conversely, when examining cellular mechanisms related to endometriosis in a particular cell type or studying direct cell-cell interactions, two-dimensional (cell lines) or three-dimensional (organoids) *in vitro* models may be more suitable (Marr *et al.*, 2025), potentially followed by the induction of endometriosis in transgenic mice.

When selecting model(s), investigators are also impacted by practical considerations. For example, endometriosis studies using human organoids or the heterologous mouse model require access to fresh human samples. Many researchers working in institutions not affiliated with hospitals specializing in endometriosis care may, therefore, lack access to fresh tissue or the necessary infrastructure to process it. As a result, these researchers rely more on cell lines, murine organoids, and the homologous mouse model to address their research questions.

Finally, all studies have time and cost limits. Not only can it take several months before a researcher has obtained enough samples for their study, but it can also take months or years to secure ethical approvals for live animal studies and to learn specific techniques for animal handling and behavioural studies. Facing funding constraints, two-dimensional cell lines can be a cost-effective choice, especially when generating preliminary data for grant submissions. When planning for rodent experiments, it is essential to factor in not only the cost of acquiring the animals, but also the expense of housing them. Organoid models have expenses associated with the specialized media required to grow and maintain organoid cultures that are often underestimated.

No single model can fully replicate the complexities of endometriosis. Thus, the bench-, clinical-, and population-based discovery supported by the five original EPHect tools needed to be enhanced by these four new EPHect experimental models SOPs. By carefully considering the research question, available resources, and practical constraints, investigators can make informed decisions about appropriate experimental models that optimize the quality, feasibility, and reproducibility of their studies.

All nine EPHect tools are freely available from https://ephect.org/, expanding the foundation for harmonized methods that ease comparison and combination of published results. Also, at https://ephect.org/, there is information on which research sites and leaders are using which specific tool(s), with the mission of facilitating and enhancing our multi-disciplinary collaborative efforts in endometriosis discovery.
